# Autologous hematopoietic stem cell transplantation improves survival outcomes in peripheral T-cell lymphomas: a multicenter retrospective real-world study

**DOI:** 10.1007/s00277-023-05416-x

**Published:** 2023-09-12

**Authors:** Meng Wu, Fengrong Wang, Shihua Zhao, Yajun Li, Wenrong Huang, Bo Nie, Haisheng Liu, Xiaoqian Liu, Wei Li, Haifeng Yu, Kun Yi, Fei Dong, Yujun Dong, Chenglu Yuan, Xuehong Ran, Xiubin Xiao, Weiping Liu, Jun Zhu

**Affiliations:** 1 Key Laboratory of Carcinogenesis and Translational Research (Ministry of Education/Beijing), Department of Lymphoma, Peking University Cancer Hospital & Institute, Beijing, China; 2 Beijing Key Laboratory of Hematopoietic Stem Cell Transplantation, Peking University People’s Hospital & Institute of Hematology, Beijing, China; 3 Department of Lymphoma and Plasmacytoma Disease, Senior Department of Hematology, the Fifth Medical Center of PLA General Hospital, Beijing, China; 4 Department of Lymphoma and Hematology, Hunan Cancer Hospital and The Affiliated Cancer Hospital of Xiangya School of Medicine, Central South University, Changsha, Hunan China; 5 Department of Hematology, the First Affiliated Hospital of Kunming Medical University, Kunming, China; 6 Department of Hematology, the Fourth Hospital of Hebei Medical University, Shijiazhuang, China; 7 Department of Hematology, The Affiliated Yantai Yuhuangding Hospital of Qingdao University, Yantai, 264000 Shandong China; 8 Department of Lymphoma, Key Laboratory of Cancer Prevention and Therapy, Sino-US Center for Lymphoma and Leukemia, Tianjin Medical University Cancer Institute and Hospital, National Clinical Research Center of Cancer, Tianjin, China; 9 Department of Lymphatic Medical Oncology, Cancer Hospital of the University of Chinese Academy of Sciences (Zhejiang Cancer Hospital), Hangzhou, China; 10 Department of Lymphoma and Hematology, Jiangxi Cancer Hospital of Nanchang University, Nanchang, China; 11 Department of Hematology, Peking University 3Rd Hospital, Beijing, China; 12 Department Hematology, Peking University First Hospital, Beijing, China; 13 Department of Hematology, Qilu Hospital (Qingdao), Cheeloo College of Medicine, Shandong University, Qingdao, China; 14 Hematology Department, Weifang People’s Hospital, Weifang, China

**Keywords:** Autologous hematopoietic stem cell transplantation, Peripheral T-cell lymphomas, Real-world study, Multicenter research

## Abstract

The aim of this study is to evaluate the survival benefit of consolidative autologous hematopoietic stem cell transplantation (ASCT) in patients with peripheral T-cell lymphomas (PTCL). In this retrospective study, the ASCT group underwent consolidative ASCT after first-line therapy at 14 transplantation centers in China between January 2001 and December 2019. Data were collected over the same time frame for the non-ASCT group from the database of lymphoma patient records at Peking University Cancer Hospital & Institute. A total of 120 and 317 patients were enrolled in the ASCT and non-ASCT groups, respectively, and their median ages were 43 years and 51 years, respectively. In the ASCT group, 101 patients had achieved complete remission (CR) and 19 patients had achieved partial remission at the time of ASCT. The median follow-up time was 40.2 months and 68 months, and the 3-year overall survival (OS) rate was 80.6% and 48.9% (*p* < 0.001) for the ASCT and non-ASCT groups, respectively. The beneficial effect of ASCT for OS remained even after propensity score-matched (PSM) analysis (81.6% vs 68.3%, *p* = 0.001). Among the 203 patients who were aged ≤ 65 years and achieved CR, ASCT conferred a significant survival benefit (3-year progression-free survival [PFS]: 67.4% vs 47.0%, *p* = 0.004; 3-year OS: 84.0% vs 74.1%, *p* = 0.010), and this was also maintained after PSM analysis (3-year PFS: 66.6% vs 48.4%, *p* = 0.042; 3-year OS: 84.8% vs 70.5%, *p* = 0.011). Consolidative ASCT improved the survival outcome of PTCL patients, even those who achieved CR after first-line therapy.

**Supplementary Information:**

The online version contains supplementary material available at 10.1007/s00277-023-05416-x.

## Introduction

Peripheral T-cell lymphomas (PTCL) are a heterogeneous group of lymphoproliferative diseases that develop from T lymphocytes. PTCL have a worse prognosis compared with B cell lymphomas, and there are fewer treatment regimens or new therapies. The clinical outcome for PTCL patients is affected by the pathological subtype. Data from the International T-Cell Lymphoma Project [1] indicate that the 5-year overall survival (OS) rate was best for patients with anaplastic lymphoma kinase-positive (ALK +) anaplastic large-cell lymphoma (ALCL) (70%), followed by ALK-negative (ALK −) ALCL (49%) and other subtypes, such as PTCL, not otherwise specified (NOS), angioimmunoblastic T-cell lymphoma (AITL), and enteropathy-associated T-cell lymphoma, which were all < 40% [1]. Thus, there is still an urgent unmet clinical need to improve the survival of patients with PTCL.

Autologous stem cell transplantation (ASCT) is one treatment option for patients with PTCL, especially those with relapsed or refractory disease [2]. However, the rate of ASCT in PTCL is low, and no prospective randomized controlled studies have been performed to demonstrate the value of ASCT as first-line consolidation therapy for PTCL. A randomized phase 3 trial compared the value of autologous and allogeneic transplantation as part of first-line therapy in PTCL, which showed the allogeneic transplantation was not superior to ASCT due to the high rate of transplant-related mortality (31%) [3]. A few single-arm prospective and retrospective studies have been performed but the survival outcomes varied greatly between studies [4–8]. The value of consolidative ASCT for PTCL, especially patients who achieved complete remission (CR) after first-line therapy (CR1), is therefore controversial [9–12]. The aim of the present study was to evaluate the benefit of first-line consolidation ASCT in patients with PTCL managed under real-world conditions. The 3-year PFS and OS rates were 64.4% and 80.6% in the patients with consolidative ASCT, while the 3-year OS rate was only 48.9% in patients without consolidative ASCT. In the young patients with CR1, ASCT also conferred a significant survival benefit in both PFS and OS (3-year PFS: 67.4% vs 47.0%, *p* = 0.004; 3-year OS: 84.0% vs 74.1%, *p* = 0.010). The results of this real-world retrospective analysis support the use of consolidative ASCT in PTCL patients after first-line therapy, even for those who achieved CR1.

## Methods

### Study participants and data collection

Data for the ASCT group were collected from consecutive PTCL patients who underwent consolidative ASCT at 14 hematology and transplantation centers in China between January 2001 and December 2019. Data for the non-ASCT group over the same time frame were collected from the database of consecutive lymphoma patients registered at Peking University Cancer Hospital & Institute. All patients had a re-review pathological diagnosis of aggressive mature T-cell lymphoma by the pathology department at each center according to the 2016 World Health Organization criteria [13] during January 2020 to December 2020. The main exclusion criteria of both two groups included patients with extranodal natural killer cell/T-cell lymphoma nasal type or primary cutaneous lymphoma and leukemia, incomplete information on initial treatment, and missing survival outcomes. Consolidative ASCT was defined as ASCT for patients who achieved CR1 or partial remission after first-line therapy (PR1). In the non-ASCT group, patients receiving consolidative ASCT were excluded. The patients did not receive ASCT mainly due to the following reasons: (a) contraindication to ASCT (such as concomitant disease or mobilization failure); (b) failure to disease remission; (c) patients’ option for economic reasons. Response criteria were defined according to the Lugano 2014 guidelines [14]. The detailed inclusion and exclusion criteria were described in the Supplements. This retrospective analysis was approved by the institutional review board of the lead institution of Peking University Cancer Hospital & Institute and all other participating institutions, and the need for patient informed consent was waived. The cutoff date for last follow-up was 31 December 2020.

### Statistical analysis

The median follow-up time for OS was estimated by the reverse Kaplan–Meier method. Progression-free survival (PFS) was calculated from the date of diagnosis to the date of disease relapse, first progression, or last follow-up, whichever occurred first. OS was calculated from date of diagnosis to the date of death from any cause or last follow-up, whichever occurred first. PFS and OS rates were estimated using the Kaplan–Meier method. Prognostic factors were analyzed using Cox regression. According to the pathological subtype, IPI score, and remission status after first-line therapy, a 1:1 propensity score-matched (PSM) analysis was carried out using the nearest-neighbor method (caliper size 0.02) to compare the survival outcomes of patients with or without ASCT. All analyses were performed using SPSS software, version 22.0 (IBM, Armonk, NY, USA).

## Results

### Clinicopathological characteristics

A total of 120 patients were included in the ASCT group. The median age was 43 years (range 14–66); 7 patients were < 18 years old, 1 patient was > 65 years old (66 years), and 6 patients were between 60 and 65 years of age. The male-to-female ratio was 2.43:1 (85 [70.8%] male). Of the 120 patients, 98 (81.6%) had advanced stage disease and 36 (30%) had at least one extralymphatic involvement. A total of 317 patients were included in the non-ASCT group. The median age was 51 years (range 14–85); 101 (31.8%) patients were > 65 years old, 232 (73.2%) patients were male, and 246 (77.6%) patients presented with advanced stage disease. Further details of the clinicopathological features are provided in Table 1.

**Table 1:** Pretherapeutic clinicopathologic patient characteristics

	ASCT group, number (%)	Non-ASCT group, number (%)
Subtype of pathology		
ALCL ALK +	38 (31.7%)	45 (14.2%)
ALCL ALK −	15 (12.5%)	42 (13.2%)
AITL	26 (21.7%)	84 (26.5%)
PTCL-NOS	30 (25.0%)	146 (46.1%)
SPTCL	4 (3.3%)	
EATL	4 (3.3%)	
Others	3 (2.5%)	
Stage		
Stage I	5 (4.1%)	23 (7.3%)
Stage II	17 (14.2%)	48 (15.1%)
Stage III	35 (29.2%)	101 (31.9%)
Stage IV	63 (52.5%)	145 (45.7%)
B symptom		
No	57 (47.5%)	143 (45.1%)
Yes	63 (52.5%)	174 (54.9%)
Extralymphatic involvements		
≤ 1	84 (70.0%)	246 (77.6%)
> 1	36 (30.0%)	71 (22.4%)
ECOG PS		
0–1	79 (65.8%)	275 (86.8%)
≥ 2	24 (20.0%)	42 (13.2%)
Unknown	17 (14.2%)	0 (0.0%)
LDH		
Normal (≤ 240 U/L)	59 (49.1%)	177 (55.8%)
Elevated(> 240 U/L)	44 (36.7%)	140 (44.2%)
Unknown	17 (14.2%)	0 (0.0%)
IPI		
0	9 (7.5%)	30 (9.5%)
1	25 (20.8%)	88 (27.8%)
2	31 (25.8%)	99 (31.2%)
3	17 (14.2%)	70 (22.1%)
4	4 (3.3%)	30 (9.5%)
Unknown	34 (28.4%)	0 (0.0%)

*ASCT*, autologous stem cell transplantation; *ALCL*, anaplastic large-cell lymphoma; *ALK* + , anaplastic lymphoma kinase expressing; *ALK − *, without anaplastic lymphoma kinase expressing; *PTCL-NOS*, peripheral T-cell lymphoma, not otherwise specified; *AITL*, angioimmunoblastic T-cell lymphoma; *SPTCL*, subcutaneous panniculitis-like T-cell lymphoma; *EATL*, enteropathy-associated T-cell lymphoma; *ECOG PS*, Eastern Cooperative Oncology Group performance status; *LDH*, lactate dehydrogenase; *IPI*, International Prognostic Index

In the ASCT group, 108 patients received first-line CHOP, CHOPE, or EPOCH regimens; 9 patients received CHOPE/EPOCH alternating with a gemcitabine-based regimen; and the remaining 3 patients received GDPE, DICE, or HyperCVAD regimens. The majority of patients (101/120, 84.2%) achieved CR1 and 19 (15.8%) achieved PR1. Notably, patients diagnosed with ALK + ALCL (38 cases) in the ASCT group all met one of the following two criteria: (a) International Prognostic Index (IPI) > 1, or (b) achieved PR1.

In the non-ASCT group, 283 patients received CHOP, CHOPE, or EPOCH regimens; 27 patients received CHOPE/EPOCH alternating with a gemcitabine-based regimen; 3 patients received HyperCVAD or ESHAP regimens; and the remaining 4 patients received an oral chemotherapy regimen. After first-line therapy, 110 patients (34.7%) in the non-ASCT group achieved CR, 82 (25.9%) achieved PR, and 125 (39.4%) had no response.

### Survival after ASCT and associated prognostic parameters

**Fig. 1:**
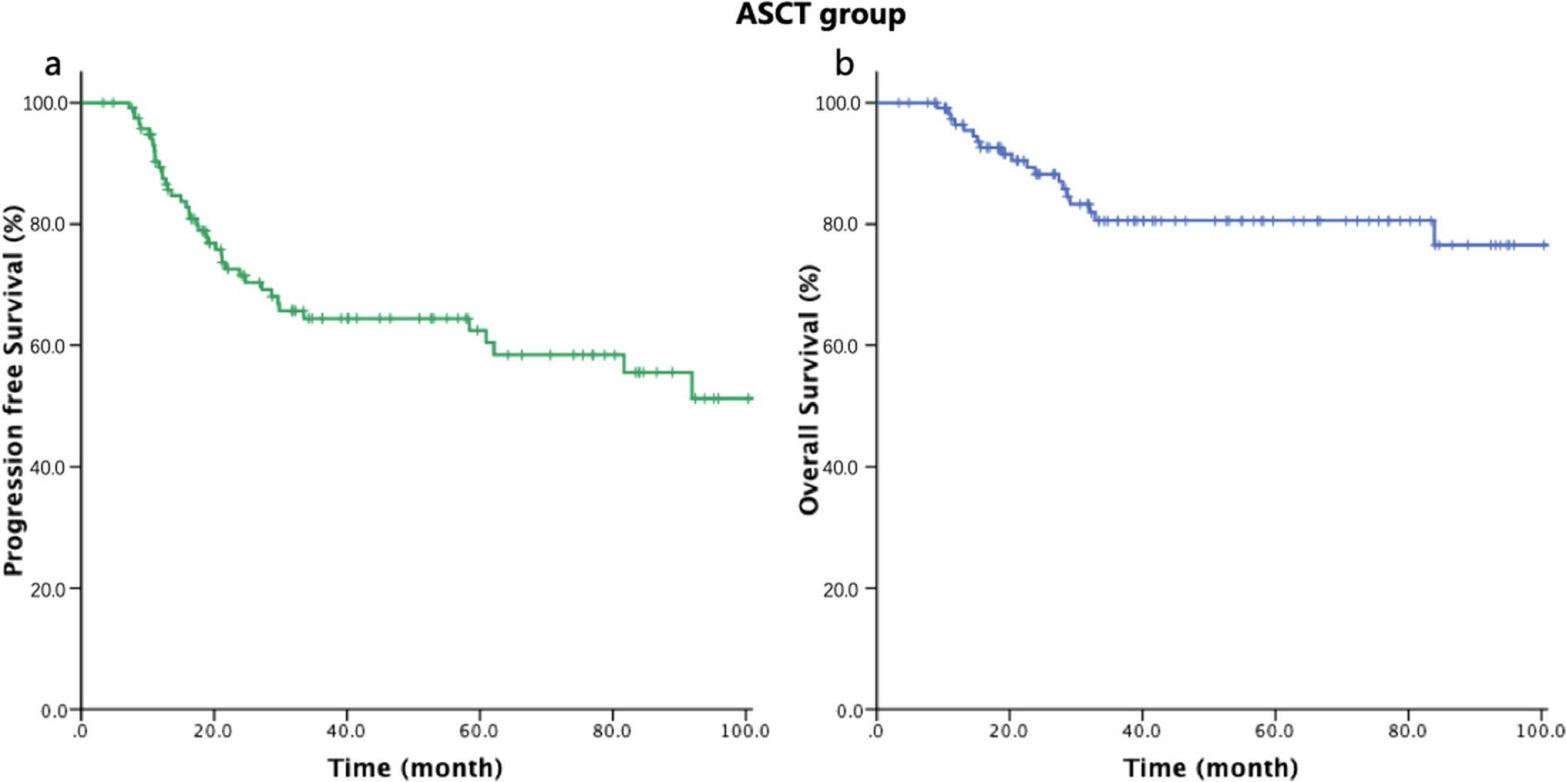
Kaplan–Meier curves for progression-free survival and overall survival of patients with autologous stem cell transplantation

In the ASCT group, the median follow-up time was 40.2 months and the 3-year PFS and OS rates were 64.4% and 80.6%, respectively (Fig. 1). There was no ASCT-related mortality. Until the last follow-up, a total of 42 patients had disease progression after HDT/ASCT, 19 of whom died as a result of progressive disease within a year. These patients with good physical conditions received chemotherapy or HDAC inhibitors as salvage therapy, or participated in clinical trials after disease progression. None of them received allogeneic hematopoietic stem cell transplantation.

**Fig. 2:**
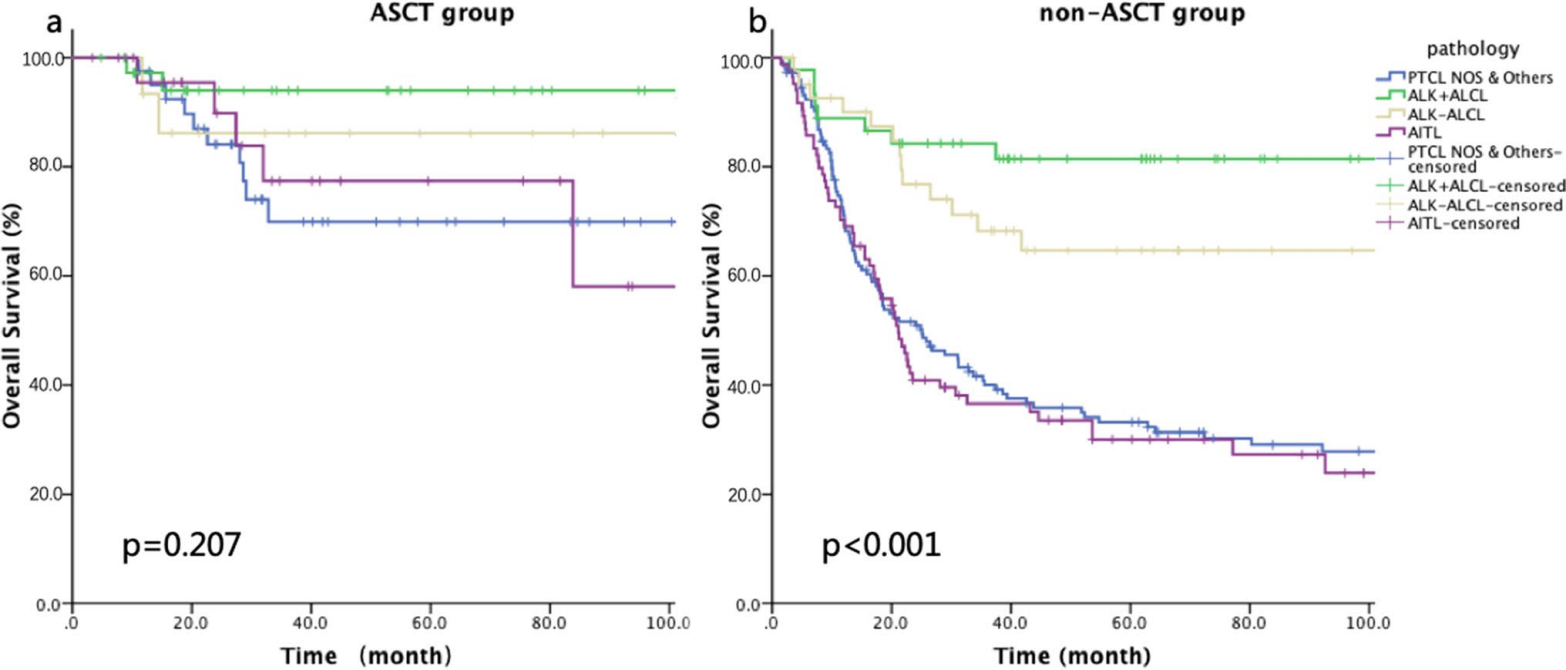
Kaplan–Meier curves for overall survival of patients in different pathological subtypes. **a** With autologous stem cell transplantation (ASCT). **b** Without ASCT

The PFS and OS rates were higher for patients who achieved CR before ASCT compared with those who achieved PR (3-year PFS: 67.4% vs 50.2%, *p* = 0.034; 3-year OS: 84.0% vs 62.9%, *p* = 0.045). Univariate analysis showed that pathological subtype was not significantly associated with the 3-year OS rate (94.0% for ALK + ALCL, 86.2% for ALK − ALCL, 77.4% for AITL, 69.9% for PTCL-NOS and other subtypes; *p* = 0.207; Fig. 2a). However, pathological subtype did correlate strongly with PFS (Table 2); specifically, patients with ALK + ALCL had significantly better 3-year PFS rates compared with patients with other subtypes. In the ASCT group, survival outcomes were not significantly associated with any of the remaining parameters evaluated, which included advanced stage, B symptoms, more than one extralymphatic involvement, poor Eastern Cooperative Oncology Group status, elevated lactate dehydrogenase before initial treatment, and intermediate or high IPI scores (2–5).

**Table 2:** Prognostic factors in univariate analysis

	*N*	3-year PFS		3-year OS	
		%	*p*	%	*p*
Subtype of pathology			0.008		0.207
ALCL ALK +	38	86.3		94.0	
ALCL ALK −	15	72.0		86.2	
AITL	26	60.8		77.4	
PTCL-NOS and others	41	46.8		69.9	
Stage			0.692		0.246
Stages I–II	22	62.9		89.5	
Stages III–IV	98	61.9		78.2	
B symptom			0.810		0.552
No	57	59.1		80.1	
Yes	63	65.8		80.8	
Extralymphatic involvements			0.535		0.479
≤ 1	84	61.8		83.4	
> 1	36	64.1		74.4	
ECOG PS			0.692		0.584
0–1	79	62.9		80.5	
≥ 2	24	61.9		84.0	
Unknown	17	-		-	
LDH			0.202		0.801
Normal (≤ 240 U/L)	59	53.4		78.7	
Elevated (> 240 U/L)	44	74.2		83.6	
Unknown	17	-		-	
IPI			0.164		0.927
0–1	36	56.6		80.4	
2–5	71	68.3		79.9	
Unknown	13	-		-	
Status before ASCT			0.034		0.045
CR	101	67.4		84.0	
PR	19	50.2		62.9	

*PFS*, progression-free survival; *OS*, overall survival; *ALCL*, anaplastic large-cell lymphoma; *ALK* + , anaplastic lymphoma kinase expressing; *ALK − *, without anaplastic lymphoma kinase expressing; AITL, angioimmunoblastic T-cell lymphoma; *PTCL-NOS*, peripheral T-cell lymphoma, not otherwise specified; *ECOG PS*, Eastern Cooperative Oncology Group performance status; *LDH*, lactate dehydrogenase; *IPI*, International Prognostic Index; *CR*, complete remission; *PR*, partial remission

### Survival of the non-ASCT group and comparison with the ASCT group

The median follow-up time for the non-ASCT group was 68 months. In this group, only 5 patients received salvage therapy and HDT/ASCT after disease progression. Other patients with relapse and refractory disease received intravenous or oral chemotherapy, novel targeted therapies, or drugs from clinical trials. None of patients received allogeneic hematopoietic stem cell transplantation.

The non-ASCT group had a significantly lower 3-year OS rate than the ASCT group (48.9% vs 80.6%, *p* < 0.001). In contrast to the ASCT group, OS of the non-ASCT group was significantly associated with pathological subtypes and IPI scores (compare Fig. 2a and b). The 3-year OS rates for patients with ALK + ALCL (82.5%) and ALK − ALCL (68.2%) were significantly higher than the rates for patients with other subtypes (36.6% for AITL, 40.0% for PTCL-NOS and other subtypes, *p* < 0.001; Fig. 2b). Similarly, an IPI score of 2–5 was highly predictive of better OS in the non-ASCT group (3-year OS: 36.4% vs 69.7% for IPI 2–5 vs 0–1; *p* < 0.001) but not in the ASCT group (*p* = 0.927).

**Fig. 3:**
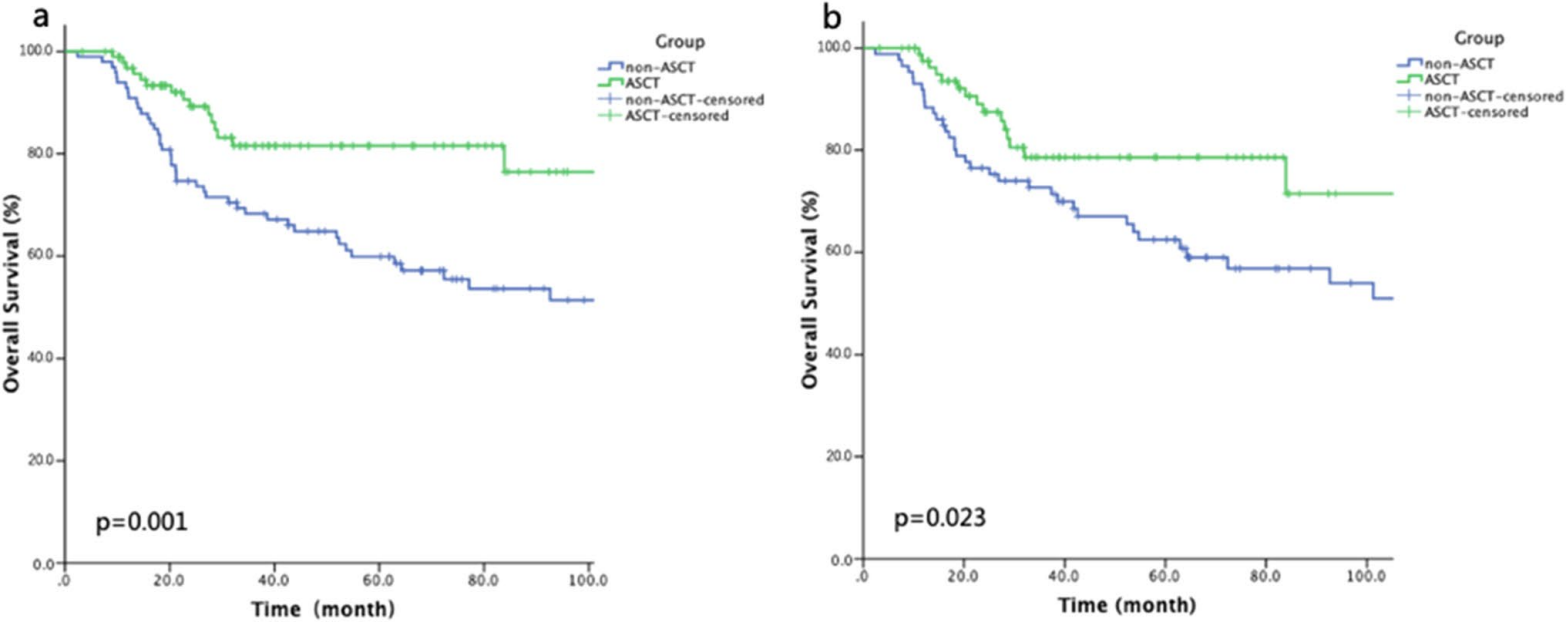
Kaplan–Meier curves for different propensity score-matched (PSM) overall survival. **a** 1:1 PSM analysis based on pathological subtype, IPI score, and remission status. **b** 1:1 PSM analysis based on pathological subtype, IPI score, remission status, and age

Considering about the discrepancies of the baseline characteristics of the two groups, 1:1 PSM analyses were carried out by controlling certain set of parameters (Fig. 3). After 1:1 PSM analysis based on pathological subtype, IPI score, and remission status after first-line therapy (*n* = 198), the benefit of ASCT for 3-year OS rate remained (81.6% vs 68.3%, *p* = 0.001; Fig. 3a), while the survival outcome was similar when the PSM analysis was based on pathological subtype, IPI score, remission status, and age (Fig. 3b). One explanation of this result is that age was not a crucial determining factor in this study, while it may also be due to the matching of IPI score which itself is a compound factor already taking the age into account.

### Survival benefit of ASCT for patients who achieved CR1

**Fig. 4:**
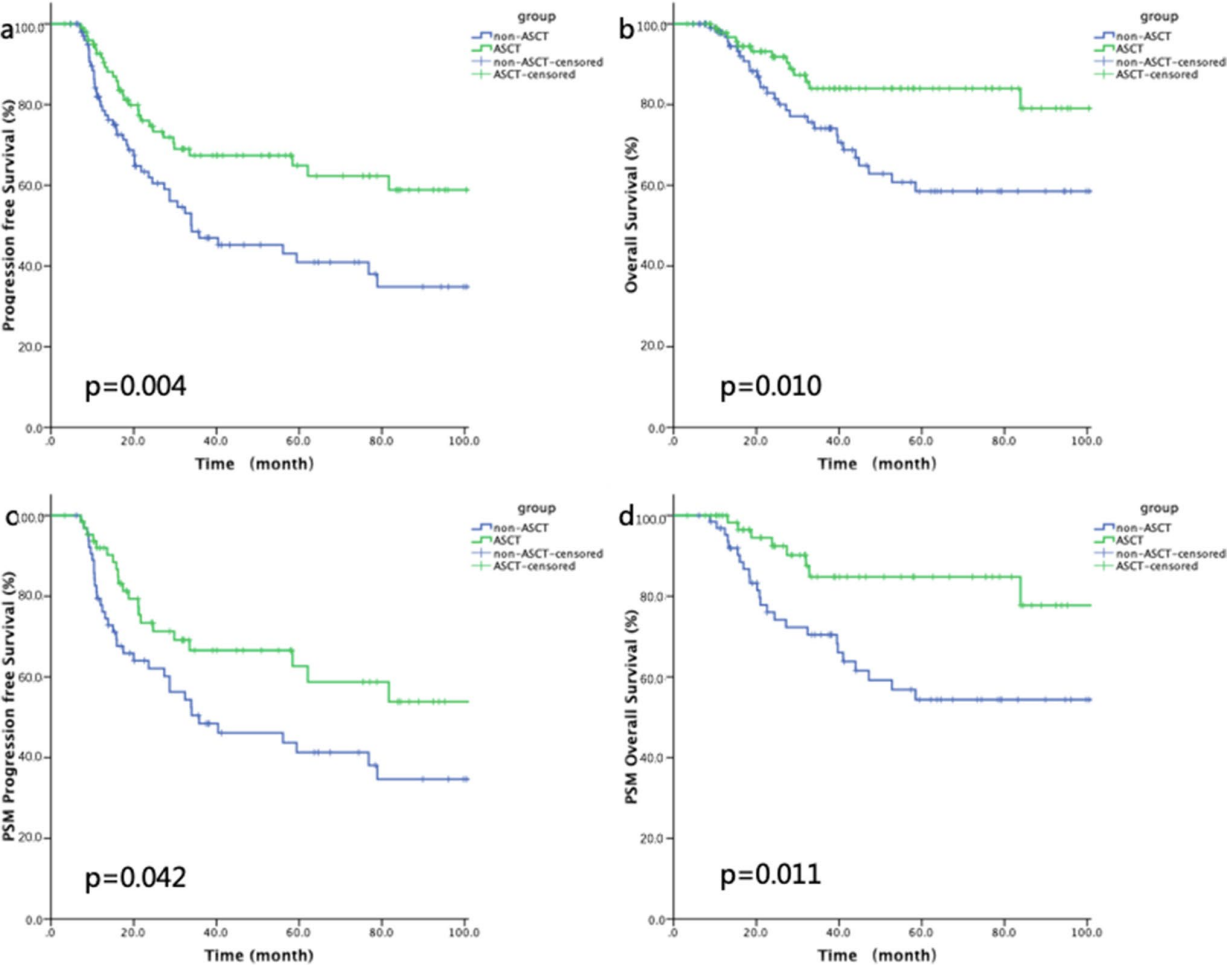
Kaplan–Meier curves for **a** progression-free survival (PFS), **b** overall survival (OS), **c** propensity score-matched (PSM) PFS, and **d** PSW OS of complete remission patients with or without autologous stem cell transplantation

We further compared the efficacy of ASCT in patients who were < 65 years of age and achieved CR1 (*n* = 101 and 102 in the ASCT and non-ASCT groups, respectively). For this group of patients, ASCT conferred a significant survival benefit (3-year PFS: 67.4% vs 47.0%, *p* = 0.004; 3-year OS: 84.0% vs 74.1%, *p* = 0.010; Fig. 4a and b). After 1:1 PSM analysis according to pathological subtype and IPI score (*n* = 130), ASCT remained beneficial for patients aged < 65 years who achieved CR (3-year PFS: 66.6% vs 48.4%, *p* = 0.042; 3-year OS: 84.8% vs 70.5%, *p* = 0.011; Fig. 4c and d).

## Discussion

The International T-Cell Lymphoma Project estimates that PTCLs account for about 15% of non-Hodgkin lymphomas (NHLs) worldwide [1], and their prevalence in China is thought to be even higher, at about 23% of NHLs [15]. A previous study from Peking University Cancer Hospital showed that the 5-year OS rate of patients with mature B cell lymphoma increased from 49% in 1996–2000 to 65% in 2011–2015 (*p* = 0.002), whereas the OS rate for patients with PTCL had improved only modestly over the past 2 decades (41% in 1996–2000, 51% in 2011–2015, *p* = 0.592) [16]. Taken together, these observations highlight the huge unmet clinical need for additional treatments for PTCL and further research on regimens that improve survival outcomes.

Here, we performed a retrospective, multicenter, real-world analysis of 120 Chinese patients with PTCL who received consolidative ASCT; this is the largest such study of Chinese patients to date. Although numerous reports have been published on the value of ASCT for PTCL, there have been no randomized controlled trials, due in large part to the diverse pathological subtypes and low incidence of PTCL. Moreover, only a few clinical studies have specifically evaluated PTCL patients who received consolidative ASCT after first-line therapy. In the NLG-T-01 study, the largest prospective study of PTCL patients (excluding ALK + ALCL patients) receiving consolidative ASCT, a total of 160 patients were enrolled and 115 completed the induction therapy with CHOEP followed by ASCT [7]. The 5-year PFS and OS rates for these patients were 44% and 51%, respectively, and pathological subtype had no significant effect on survival rates. In a prospective study conducted in Germany [17] of 111 patients with PTCL (excluding ALK + ALCL), 75 patients achieved either CR or PR after 4–6 cycles of CHOP and ASCT. The 5-year PFS and OS rates for these patients were 39% and 44%, respectively. All other prospective and retrospective studies of PTCL with consolidative ASCT have enrolled a relatively limited number of patients. A systematic review/meta-analysis [18] showed that the pooled PFS and OS rates in the prospective studies were 32.8% and 53.8%, respectively, and those in the retrospective studies were 55% and 67.9%, respectively, after ASCT. In our study, the 3-year PFS and OS rates in the ASCT group (*n* = 120) were 64.4% and 80.6%, respectively, which was a satisfactory survival outcome.

Previous studies have shown an association between survival outcomes and pathological subtype and IPI score at diagnosis in PTCL [1, 19]. In the present study, we found that the non-ALCL subtype and IPI score 2–5 were associated with poor OS in the non-ASCT group, whereas neither parameter correlated with OS in the ASCT group. PSM analysis revealed that the OS rate was higher for the ASCT group compared with the non-ASCT group even among patients with similar pathological subtypes and IPI scores. This finding indicates that sensitivity to chemotherapy was a more important favorable prognosis factor than baseline characteristics such as pathological subtype and IPI score.

The value of consolidative ASCT in PTCL is controversial, especially for patients who achieved CR1. The Swedish Lymphoma Registry study showed that ASCT conferred a significant survival advantage (5-year PFS: 41% vs 20%, 5-year OS: 48% vs 26%, *p* < 0.01) [20]; in contrast, some meta-analyses and multicenter retrospective reviews found no significant survival benefit of ASCT in PTCL [10, 21]. In the COMPLETE study conducted in the USA, the survival of 119 PTCL patients who achieved CR1 was followed; of these, 36 received ASCT and 83 did not [12]. This study found that the median OS and PFS were longer in the ASCT group compared with the non-ASCT, but the differences were not statistically significant (OS: not reached vs 57.6 months, *p* = 0.06; PFS: 57.6 months vs 47.5 months, *p* = 0.23). In the present study, we observed a significant survival advantage conferred by ASCT in CR1 patients < 65 years of age. These results support the use of ASCT as a reasonable option for consolidation after CR1 to increase survival. There was an ongoing randomized controlled study evaluating the role of consolidative ASCT in PTCL with CR1 (NCT05444712).

In addition to ASCT, frontline allogeneic SCT has also been explored in several studies of PTCL patients. A randomized phase 3 trial compared ASCT to allo-SCT for consolidation treatment [3] and found no significant differences in PFS or OS between the treatment arms, whether the intent-to-treat population or the transplant recipients were evaluated, after a median follow-up of 42 months. However, the relapse rates were 0% and 36% and the treatment-related mortality rates were 31% and 0% in the allo-SCT and ASCT groups, respectively. Data from the MD Anderson Cancer Center (Houston, TX, USA) showed similar results [22]. Considering the efficiency and safety of ASCT compared with allo-SCT, these observations suggest that ASCT should remain the preferred option for younger patients with PTCL.

## Conclusion

In conclusion, the results of this retrospective analysis support the use of consolidative ASCT to improve survival outcomes of PTCL patients after first-line therapy, even for those who achieved CR1. In the absence of high-quality data from randomized trials, the findings reported here may help to streamline the decision-making process for treatment of PTCL patients. However, there is a clear need for prospective randomized, controlled clinical trials to determine the optimal treatment strategies for PTCL.

### Supplementary Information

Below is the link to the electronic supplementary material.

Supplementary file1 (DOCX 16 KB)

## Data Availability

The data that support the findings of this study are available on request from the corresponding author. The data are not publicly available due to privacy or ethical restrictions.
