# Supplementary material for: Autologous hematopoietic stem cell transplantation improves survival outcomes in peripheral T-cell lymphomas: a multicenter retrospective real-world study
Source: Ann Hematol. 2023 Sep 12;102(11):3185–93. doi: 10.1007/s00277-023-05416-x (PMC10567887; doi:10.1007/s00277-023-05416-x)
Supplement: Supplementary file 1 — Supplementary file1 (DOCX 16 KB) [file 277_2023_5416_MOESM1_ESM.docx]

Supplements

1. Inclusion and exclusion criteria

1.1 Inclusion Criteria for autologous stem cell transplantation (ASCT) group

- Histologically proven aggressive mature T-cell lymphoma, including anaplastic large-cell lymphoma (ALK+ and ALK-), peripheral T-cell lymphoma not otherwise specified, angioimmunoblastic T-cell lymphoma, subcutaneous panniculitis-like T-cell lymphoma, enteropathy-associated T-cell lymphoma, monomorphic epitheliotropic intestinal T-cell lymphoma, intestinal T-cell lymphoma NOS, subcutaneous panniculitis-like T-cell lymphoma, and hepatosplenic T-cell lymphoma.
- Received systematic first-line intravenous therapy.
- Achieved complete remission or partial remission after first-line therapy.
- Received consolidative ASCT after first-line therapy.
- Diagnosis and ASCT date between January 2001 and December 2019

1.2 Exclusion Criteria for ASCT group

- Histologically proven extranodal natural killer cell/T-cell lymphoma nasal type, primary cutaneous lymphoma, and leukemia.
- Received consolidative allogeneic transplantation after first-line therapy.
- Patients without complete initial treatment information and survival data.

1.3 Inclusion Criteria for non-ASCT group

- Histologically proven aggressive mature T-cell lymphoma, including anaplastic large-cell lymphoma (ALK+ and ALK-), peripheral T-cell lymphoma not otherwise specified, angioimmunoblastic T-cell lymphoma, subcutaneous panniculitis-like T-cell lymphoma, enteropathy-associated T-cell lymphoma, monomorphic epitheliotropic intestinal T-cell lymphoma, intestinal T-cell lymphoma NOS, subcutaneous panniculitis-like T-cell lymphoma, and hepatosplenic T-cell lymphoma.
- Received systematic first-line intravenous therapy.
- Diagnosis date between January 2001 and December 2019

1.4 Exclusion Criteria for non-ASCT group

- Histologically proven extranodal natural killer cell/T-cell lymphoma nasal type, primary cutaneous lymphoma, and leukemia.
- Received consolidative ASCT after first-line therapy.
- Received consolidative allogeneic transplantation after first-line therapy.
- Patients without complete initial treatment information and survival data.
